# Development of New Family Caregiving Motivation Scale

**DOI:** 10.1093/geroni/igaf122.1484

**Published:** 2025-12-31

**Authors:** Dingyue Wang, Qing Yang, Michael Cary, Tamryn Gray, Cristina Hendrix

**Affiliations:** Duke University, Seattle, Washington, United States; Duke University, Durham, North Carolina, United States; Duke University, Durham, North Carolina, United States; The University of North Carolina at Chapel Hill, Chapel Hill, North Carolina, United States; Duke University, Durham, North Carolina, United States

## Abstract

Caregiver motivation refers to the underlying reasons individuals choose to take on or continue in the role of a family caregiver for those with chronic illnesses or disabilities. Motivation for caregiving varies among individuals. Some naturally embody the role of a family caregiver or feel a strong calling to take on this responsibility, while others may not find caregiving to be aligned with their personality or strengths. Existing measures do not fully capture the multifaceted nature of caregiving motivation. This study aimed to develop and validate the Caregiver Motivation Scale (CMS). A total of 227 family caregivers were recruited through Amazon CloudResearch’s supervised panel. The 13-item CMS was developed based on concept analysis, an in-depth qualitative study (caregiver interviews, n = 20), and an expert panel (n = 4). Validity was assessed using the Old Caregiver Motive Scale (for convergent validity) and the Caregiver Preparedness Scale (for divergent validity). Items with a factor loading above 0.45 were retained. Principal Axis Factoring identified two distinct factors: (1) Intrinsic motivation (e.g., love, emotional connection, appreciation, sense of purpose) and (2) Obligatory motivation (e.g., external expectations, duty, responsibility, lack of choice). Nine items loaded onto Factor 1, and four onto Factor 2. The CMS demonstrated good psychometric properties (S-CVI = 1, Cronbach’s alpha = 0.764, Kaiser’s MSA = 0.875, RMSR = 0.046) and serves as a valid and reliable measure of caregiver motivation, distinguishing between intrinsic and obligatory motivations. Future research should explore its use in longitudinal studies and diverse caregiving populations.

